# Covalent Immobilization of Organic Photosensitizers on the Glass Surface: Toward the Formation of the Light-Activated Antimicrobial Nanocoating

**DOI:** 10.3390/ma14113093

**Published:** 2021-06-04

**Authors:** Aleksandra Nyga, Dominika Czerwińska-Główka, Maciej Krzywiecki, Wioletta Przystaś, Ewa Zabłocka-Godlewska, Sebastian Student, Monika Kwoka, Przemysław Data, Agata Blacha-Grzechnik

**Affiliations:** 1 Faculty of Chemistry, Silesian University of Technology, Strzody 9, 44-100 Gliwice, Poland; aleksandra.nyga@polsl.pl (A.N.); dominika.czerwinska-glowka@polsl.pl (D.C.-G.); przemyslaw.data@polsl.pl (P.D.); 2 Center for Science and Education (CSE), Institute of Physics, Silesian University of Technology, Konarskiego 22B, 44-100 Gliwice, Poland; maciej.krzywiecki@polsl.pl; 3 Faculty of Energy and Environmental Engineering, Silesian University of Technology, 44-100 Gliwice, Poland; wioletta.przystas@polsl.pl (W.P.); ewa.zablocka-godlewska@polsl.pl (E.Z.-G.); 4 Biotechnology Centre, Silesian University of Technology, 44-100 Gliwice, Poland; sebastian.student@polsl.pl; 5 Faculty of Automatic Control, Electronics and Computer Science, Silesian University of Technology, 44-100 Gliwice, Poland; monika.kwoka@polsl.pl; 6 Institute of Electronics, Silesian University of Technology, Akademicka 16, 44-100 Gliwice, Poland

**Keywords:** chemical grafting, photoactive layer, antimicrobial coating, reactive oxygen species (ROS), phenothiazines, porphyrins

## Abstract

Two highly efficient commercial organic photosensitizers—azure A (AA) and 5-(4-aminophenyl)-10,15,20-(triphenyl)porphyrin (APTPP)—were covalently attached to the glass surface to form a photoactive monolayer. The proposed straightforward strategy consists of three steps, i.e., the initial chemical grafting of 3-aminopropyltriethoxysilane (APTES) followed by two chemical postmodification steps. The chemical structure of the resulting mixed monolayer (MIX_TC_APTES@glass) was widely characterized by X-ray photoelectron (XPS) and Raman spectroscopies, while its photoactive properties were investigated in situ by UV–Vis spectroscopy with α-terpinene as a chemical trap. It was shown that both photosensitizers retain their activity toward light-activated generation of reactive oxygen species (ROS) after immobilization on the glassy surface and that the resulting nanolayer shows high stability. Thanks to the complementarity of the spectral properties of AA and APTPP, the effectiveness of the ROS photogeneration under broadband illumination can be optimized. The reported light-activated nanocoating demonstrated promising antimicrobial activity toward *Escherichia coli* (*E. coli*), by reducing the number of adhered bacteria compared to the unmodified glass surface.

## 1. Introduction

Hospital-acquired infections, also called nosocomial infections, happen to more than 8.0% of patients and result in approximately 37k and 99k deaths each year in Europe and the USA, respectively, as stated by the World Health Organization (WHO). The medical complications due to these nosocomial infections may cause serious health problems and/or prolonged stay in hospitals, which also has an economic impact on the healthcare system [1]. Moreover, excessive use of antibiotics results in the decrease of their effectiveness, which in turn will lead to the development of a generation of pathogens resistant even to novel-class antimicrobial medicines [2]. Nowadays, due to the COVID-19 pandemic, even more attention is focused on the sterilization of various surfaces mainly, but not only, in the healthcare-related areas. One of the possible strategies is the application of the antimicrobial coatings on surfaces [3]; these can be generally divided into passive ones (lowering bacterial adhesion, such as poly(ethylene glycol)) and active materials with silver or copper nanoparticles, quaternary ammonium salts, cations of fluorinated polymers, etc. [4,5]. Lately, the light-activated layers that can produce reactive oxygen species (ROS) are being intensely investigated, since ROS show tremendous antibacterial, antiviral, and antifungal properties [6]. ROS, such as singlet oxygen or superoxide radicals, have been studied for more than 50 years now [7]. Their cytotoxicity against microbes is a base of photodynamic antimicrobial chemotherapy (PACT). ROS are highly effective against microorganisms, and they act in a nonselective way (e.g., oxidation of enzymes, increase in ions’ cell-wall permeability, etc.). The ability to denature antioxidant enzymes is specific to the singlet oxygen molecule [8,9,10,11,12].

The modification of the surfaces with photoactive compounds can be done through either physical or chemical methods [13]. The physical ones, which result in the noncovalent binding, are typically based on the deposition of a matrix, e.g., resin or polymer [14], which acts as a carrier for the photoactive substances, or on the physical adsorption of the photosensitizer molecule [2,15]. In the first case, cellulose acetate or polyurethanes, e.g., have been used as a polymeric matrix for the photosensitizers, and the resulting layers showed high antimicrobial activity against *Staphylococcus aureus, Escherichia coli, Clostridium difficile, Candida albicans,* and *Pseudomonas aeruginosa* [2,3,6,9,15,16,17,18,19,20,21,22]. However, such coatings usually possess limited long-term stability, due to various factors, e.g., bleaching. This can be overcome by forming a covalent bond between the photosensitizer molecule and the surface atoms. One of the most popular methods allowing for the chemical immobilization of the (photoactive) organic molecules is grafting [3,23,24,25,26] or, in the case of conductive surfaces, electrografting [27,28]. The main advantage of this process is the formation of a strong covalent bond between the immobilized molecule and surface atoms resulting in higher stability of the thin films, which is crucial for their practical use.

**Scheme 1:**
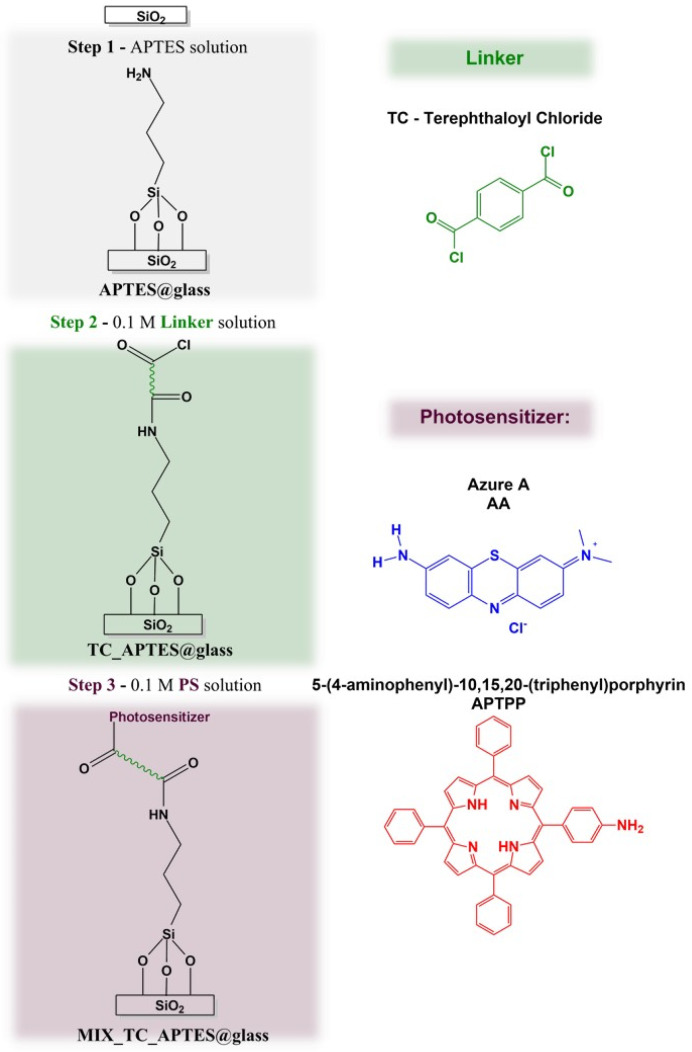
General scheme of the chemical grafting of the light-activated mixed monolayer on the glass surface.

The formation of the antimicrobial layers on glassy surfaces seems an important issue since glass is an indispensable element of the equipment of operating rooms, wards, and personal electronics (windows, glass surfaces of light sources, monitor screens or phone screens) and, thus, can be a great source of pathogens, causing nosocomial infections. Thus, in this work, two commercially available organic photosensitizers (PS)—Azure A (AA) and 5-(4-aminophenyl)-10,15,20-(triphenyl)porphyrin (APTPP) (Scheme 1)—were immobilized on the glass surface, in order to form a light-activated antimicrobial nanocoating. The proposed straightforward deposition strategy, yielding the photoactive layer covalently bound to the surface, consists in the grafting of 3-aminopropyltriethoxysilane (APTES) and the consecutive postfunctionalization of the resulting monolayer based on the reactivity of the primary amino groups present in the APTES structure [29,30]. Importantly, since AA and APTPP photosensitizers show complementary spectral properties, the deposited mixed monolayer is characterized by broadband absorbance, which is crucial to get high efficiency of the ROS photogeneration under daylight. The structure of the monolayer was confirmed using spectroscopic methods. The light-activated ROS production was tested with α-terpinene. The preliminary investigations of the antimicrobial properties of the presented MIX_TC_APTES@glass were done against the Gram-negative strain of *Escherichia coli* (*E. coli*). The scanning electron microscopy (SEM) enabled to assess the density of bacterial cells, and their viability was exhibited via fluorescent confocal microscopy.

## 2. Materials and Methods

### 2.1. Materials

Azure A (AA) (Fluka, Charlotte, NC, USA, purity >90%) and 5-(4-aminophenyl)-10,15,20-(triphenyl)porphyrin (APTPP, purity >98%, PorphyChem, Dijon, France) (Scheme 1) were selected for the formation of a photoactive layer on the glass surface. 3-Aminopropyltriethoxysilane (APTES, purity 99%), used for the chemical grafting of glass, was obtained from Acros Organics (Geel, Belgium), while terephthaloyl chloride (TC, purity ≥99%, Sigma Aldrich, Darmstadt, Germany) was used as a linker molecule. Triethylamine, analytical grade, was purchased from POCh, Gliwice, Poland. The reactive oxygen species trap—α-terpinene (purity >90%)—was obtained from TCI (Tokyo, Japan). The organic solvents, tetrahydrofuran (99.5%), toluene (99.85%), isopropanol (99.5%), ethanol (99.8%), and acetone (95%), were obtained from Acros Organics (Geel, Belgium). Acetonitrile was purchased from Honeywell (Charlotte, NC, USA) (≥99.9%). Sodium hydroxide (Acros Organics, Geel, Belgium, 1N standard solution) and hydrochloric acid (Chempur, Piekary Slaskie, Poland, 35–38% pure per analysis) were used for pretreatment of the glass substrates. The antimicrobial activity of MIX_TC_APTES@glass surface was examined using a bacterial strain of Gram-negative *Escherichia coli* (DSMZ, 30083, U5/41), which was cultivated in 23 g/L agar broth (BTL, Warsaw, Poland) at 35 °C for 24 h in an incubator. Subsequently, a suspension of bacteria (10.5 × 10^8^ CFU/mL according to the McFarland scale) in physiological saline (0.85% water solution of NaCl) (Acros Organics, Geel, Belgium) was prepared. Glutaraldehyde (Fisher Bioreagents, Waltham, MA, USA) was used for the SEM imaging of the bacteria.

### 2.2. Formation and Characterization of the Photoactive Layer

#### 2.2.1. Glass Substrate Pretreatment 

The glass pretreatment procedure was done according to the literature [31,32]. Firstly, the substrates were sonicated in an ultrasound cleaner in acetone for 15 min and then in deionized water also for 15 min. Further, the glasses were soaked in 1 M NaOH in a Teflon beaker for an hour. After re-sonication in deionized water for 15 min, the substrates were placed in 36% HCl for an hour. After that, the glass was re-sonicated in deionized water for 15 min. In the end, the substrates were sonicated in isopropanol.

#### 2.2.2. Photoactive Monolayer Formation 

The selected photosensitizers were bound to the glass surface in the three-step process (Scheme 1). In the first step, the freshly prepared glassy slides were immersed in 10% APTES solution in toluene for 24 h at room temperature [31,32,33,34,35,36]. The resulting APTES@glass substrates were copiously rinsed with toluene. In the second step, the freshly deposited layer was postmodified by immersion in 0.1 M TC/THF solution with the few drops of triethylamine. After 24 h, the modified glass slides (TC_APTES@glass) were taken out and rinsed with THF. In the final step, linker_APTES@glass surfaces were immersed in 0.1 M PS/THF (PS: AA, APTPP, or AA + APTPP) with a few drops of triethylamine for 24 h. The resulting AA_TC_APTES@glass, APTPP_TC_APTES@glass, and MIX_TC_APTES@glass layers were copiously rinsed with THF, methanol, and water to remove any weakly adsorbed species.

#### 2.2.3. X-ray Photoelectron Spectroscopy 

X-ray photoelectron spectroscopy (XPS) was done using the PREVAC (Rogów, Poland) EA15 hemispherical electron energy analyzer with the 2D multichannel plate detector and Al-Kα X-ray source (PREVAC dual-anode XR-40B source, 1486.60 eV). The system base pressure was equal to 9 × 10^−9^ Pa. Pass energy equal to 200 eV (scanning step 0.9 eV) or 100 eV (scanning step 0.05 eV) was set for survey spectra and for high-resolution spectra, respectively. For the charging effect compensation, the electron flood gun was applied working in a low-energy range (in order to avoid electron-beam-induced degradation effects). The binding energy scale was calibrated with respect to C–C component of C1s spectra (284.8 eV) [37]. The acquired spectra were fitted using CASA XPS^®^ software (version 2.3, Cheshire, UK).

#### 2.2.4. Raman Spectroscopy 

Raman spectra of both the photosensitizers, i.e., AA and APTPP; MIX_TC_APTES@glass; and the unmodified glass substrate were registered using a Renishaw inVia Raman Microscope (Renishaw, Inc., New Mills, UK), 514 nm diode excitation laser, and 2400 lines/mm grating. The Renishaw software (WiRE version 3.2, New Mills, UK) was used for smoothing and baseline subtraction.

### 2.3. Photogeneration of Reactive Oxygen Species (ROS)

The ability of the prepared layers to generate singlet oxygen was tested with α-terpinene in acetonitrile (0.05 mM solution). A modified glass plate was placed in a 10 × 4 mm^2^ quartz cuvette (Hellma Analytics, Müllheim, Germany) and illuminated either with a xenon lamp or with a diode laser. The xenon lamp was used as a broadband illumination source. In contrast, 445 and 638 nm diode lasers (Oxxius, Lannion, France, LBX-445-100CSB-PP and LBX-638-150-ELL-PP) were used as a light source exciting specifically APTPP or AA, respectively. The light-activated ROS generation was followed in situ using a Hewlett Packard (Palo Alto, CA, USA) 8452A UV–Vis spectrometer. The course of the investigated process was observed by the drop in the absorbance of α-terpinene at 266 nm.

### 2.4. Microbiological Analysis

#### 2.4.1. Bacterial Strain and Culture Conditions 

Both MIX_TC_APTES@glass and unmodified glass slides were sterilized by immersion in 70% ethanol in a sterile 12-well plate for an hour in the dark. In the next step, the well plates were washed three times with distilled water and left to dry in the dark. The bacterial suspension (0.1 mL) in culture medium (2 mL), composed of 10 g/L tryptone (BTL), 5 g/L yeast extract (BTL), and 10 g/L NaCl, pH = 7, was dropped on the sterilized samples. The bacterial culture was carried out for 48 h; for the whole time, samples were exposed to conventional indoor lighting (fluorescent ceiling lamp with two 60 watts bulbs). The analysis of bacteria was performed at two time points, i.e., at 3 and 48 h of culture. To assess the reliability and repeatability of the results, all the experiments were conducted three times, and the mean value of the results (with ± standard deviation) was presented. The statistical significance (*p* < 0.05) was determined with the ^t^-test.

#### 2.4.2. Bacterial Cell Staining and Imaging 

To assess the viability of bacteria on selected surfaces of glass and MIX_TC_APTES@glass, the LIVE/DEAD^®^ BacLight Bacterial Viability Kit (Life Technologies, Thermo Fisher Scientific, Waltham, MA, USA) assay was done. Live or dead bacterial cells were labeled in green (SYTO9 stain) or in red (D-propidium iodide), respectively. Confocal fluorescence microscopy (Olympus FluoView FV1000, FV1000, Tokyo, Japan) was applied in the visualization and analysis of the percentage of living and dead bacterial cells. The image analysis was performed with ImageJ (NIH, Bethesda, MD, USA) software. In the case of SEM (Phenom ProX) analysis, *E. coli* on the substrates were fixed with 3% glutaraldehyde solution during 24 h, following by the washing with the distilled water and exposition to ethanol having concentration equal to 30%, 50%, 70%, 80%, 90%, 95%, and 99.8%, for 10 min. Then, the samples were dried in the oven (24 h, 50 °C) and they were sputtered-coated with a thin gold film for 20 min at 20 mA (Q150R Quorum Technologies). The images were acquired using following parameters: accelerating voltage—15 kV and magnification—5000×. The average density, given by the number of bacterial cells/200 µm^2^, was determined with the ImageJ 1.8.0 (NIH, Bethesda, MD, USA) software.

## 3. Results and Discussion

### 3.1. Chemical Grafting and Spectroscopic Characterization of the Photoactive Mixed Monolayer (MIX_TC_APTES@glass)

#### 3.1.1. Chemical Grafting and XPS Spectroscopy

For the formation of the grafted mixed monolayer, a three-step procedure was applied, shown in Scheme 1. The chemical structures of the deposited layers were checked after each modification step by XPS spectroscopy. The analysis of the high-resolution XPS spectra was done using CASA XPS software with the Shirley function applied for background subtraction and the Gaussian (70%)–Lorentzian (30%) lines product for the component’s representation.

**Figure 1:**
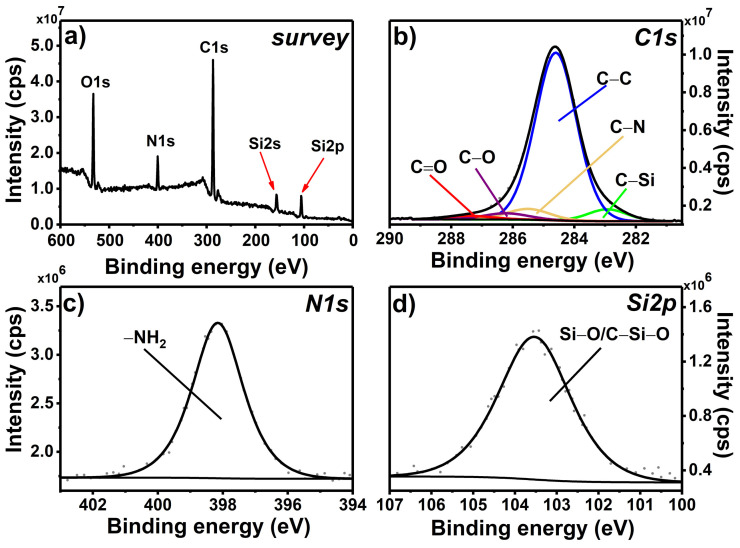
XPS survey spectrum (**a**) and high-resolution spectra of C1s (**b**), N1s (**c**), and Si2p (**d**) regions registered for the APTES@glass monolayer.

In the first step, the APTES molecule was chemically grafted on the glass surface to form a self-assembled monolayer [35]. A nonpolar environment was used in order to reduce the possibility of the formation of the hydrogen bonding between –NH_2_ present in APTES and OH groups present at the glass surface [33,36]. In the survey scan recorded for APTES@glass (Figure 1a), O1s, N1s, C1s, and Si2p peaks are observed at approximately 530, 400, 285, and 102 eV, respectively. While the presence of oxygen, carbon, and silicon signals is due to both APTES and the glass substrate, the nitrogen N1s peak is specific to the grafted organic layer.

The decomposition of the high-resolution C1s spectrum (Figure 1b) shows five components at 282.9, 284.8, 285.5, 286.2, and 287.5 eV attributed to C–Si, C–C, C–N, C–O, and C=O, respectively. C–N and C–Si components, being in the ratio close to 1, are characteristic for the APTES monolayer [33,34,38], while C–C and C–O come from the grafted organic layer and the adventitious carbon residues [38]. Low content of the C–O component, approximately 3.4%, suggests that almost all –OC_2_H_5_ groups present in the APTES molecule are involved in the grafting process, forming a covalent bond either with the glass surface atoms or with each other [33].

The analysis of the N1s region (Figure 1c) reveals the presence of one component at 398.2 eV that can be assigned to –NH_2_ group present in the APTES molecule bound to the glass surface. Importantly, no signal at approximately 402 eV was observed, indicating that neither –NH_3_^+^ nor –NH_2_/OH hydrogen bonding was formed during the SAM deposition [33,38]. This is of particular importance, since the consecutive postmodification steps are based on the accessibility of the “free” amino groups. Finally, only one asymmetric component at 103.5 eV was observed for the Si2p high-resolution spectrum (Figure 1d). The major contribution to this region is by Si–O component originating from glass substrate. The contribution from the examined layer is represented by asymmetry (low binding energy side) and by region energy shift from the expected 104.0 eV for the clean glass to 103.5 eV due to the presence of the C–Si–O component [33,35].

**Figure 2:**
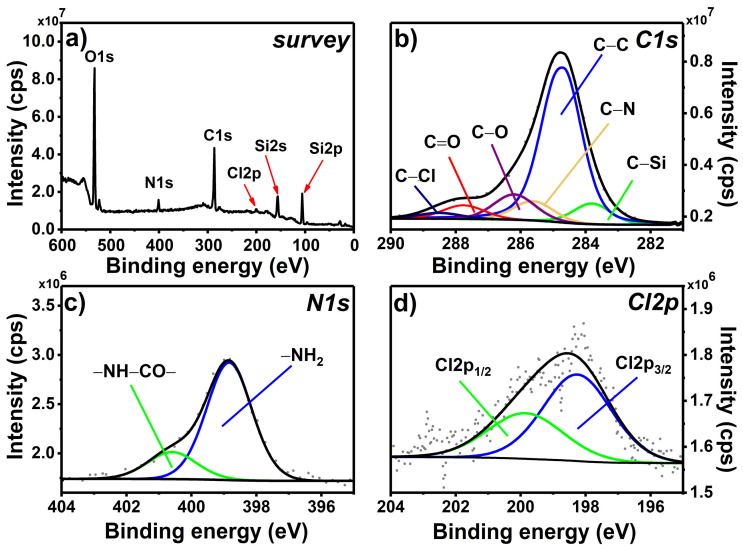
XPS survey spectrum (**a**) and high-resolution spectra of C1s (**b**), N1s (**c**), and Cl2p (**d**) regions registered for the TC_APTES@glass monolayer.

The chemically grafted APTES monolayer was, in the next step, modified in the consecutive chemical reactions. The first step of postfunctionalization was based on the reactivity of the primary amine present in the APTES@glass layer. Thus, the slides were immersed in a solution of diacyl chloride (TC linker) in order to form an amide group (Scheme 1, Step 2). The XPS survey spectrum of the resulting TC_APTES@glass coating (Figure 2a) reveals the presence of the peaks that occurred also in the APTES@glass spectrum, i.e., O1s, N1s, C1s, and Si2p spectral lines at approximately 530, 400, 285, and 102 eV, with an additional signal of chlorine—Cl2p at 199 eV. The presence of the latter is also visible in the C1s high-resolution spectrum (Figure 2b) as the C–Cl component occurring at 288.5 eV from acyl chloride groups in TC linker. The other components previously observed in the C1s region for APTES@glass, i.e., C–Si, C–C, C–N, C–O, and C=O, are also observed in this case, though in slightly different ratios.

The most significant changes can be observed in the N1s high-resolution spectrum (Figure 2c). In the case of TC_APTES@glass, two clearly visible components can be distinguished: –NH_2_ at 398.8 eV and –NH–CO– at 400.6 eV [38], which confirms the occurrence of the reaction between surface-bound –NH_2_ groups and –COCl groups in the TC-linker molecule. The presence of the unreacted amine units is probably related to the steric hindrance of this surface-based reaction. The percentage contribution of –NH–CO– component to N1s signal is in agreement with the C–Cl/C–N ratio in C1s. The analysis of the Cl2p region (Figure 2d) confirms that only one component, with its spin-orbit splitting counterpart, is observed—Cl2p_3/2_ at 198.3 eV, which can be assigned to chlorine present in the unreacted acyl chloride groups in TC-linker. The ratio of –NH–CO– component in N1s region and Cl2p_3/2_ component in Cl2p region is close to 1 (taking into account the corresponding relative sensitive factors), indicating that only one –COCl group (per TC molecule) takes part in the reaction with the amine group, while the other –COCl group remains “available” for the consecutive functionalization.

**Figure 3:**
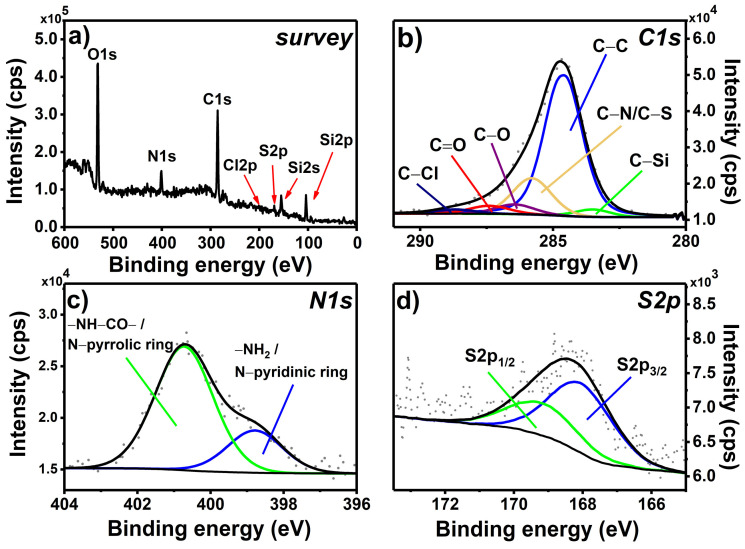
XPS survey spectrum (**a**) and high-resolution spectra of C1s (**b**), N1s (**c**), and S2p (**d**) regions registered for the MIX_TC_APTES@glass monolayer.

The last step of the formation of the photoactive layer was, as seen in Step 2, based on the reactivity of the acyl chloride and the amine group. Here, the remaining unreacted acyl chloride groups bound to the glass surface (TC_APTES@glass, Scheme 1) were reacted with the primary amine groups present in the selected photosensitizers, i.e., AA and APTPP. The recorded survey spectrum of MIX_TC_APTES@glass (Figure 3a) reveals the presence of the same characteristic signals as for the TC_APTES@glass sample with an additional signal of S2p at approximately 168 eV. The decomposition of the C1s high-resolution spectrum (Figure 3a) shows the presence of six components: C–Si, C–C, C–N/C–S, C–O, C=O, and C–Cl at 283.5, 284.8, 285.8, 286.4, 287.4, and 288.6 eV, respectively. The significantly higher contribution of the C–N/C–S component can be observed, which is related to the attachment of phenothiazine (AA) and porphyrin (APTPP) rings. Moreover, the decrease in the content of the C–Cl component compared to the C1s spectrum of TC_APTES@glass, indicates the breakage of the C–Cl bond present in the attached TC linker in Step 3 due to the formation of the amide bond in the reaction between acyl chloride of TC and primary amine present in AA or APTPP.

Clear changes can be also observed in the N1s region recorded for the MIX_TC_APTES@glass sample (Figure 3b)—two components can be distinguished, located at 398.8 and 400.7 eV, that can be assigned to –NH_2_/N-pyridinic ring and –NH–CO–/N-pyrrolic ring, respectively. The first one is present in the phenothiazine structure, i.e., AA photosensitizer, while the second one is found in porphyrin, APTPP [39,40], which together confirms the efficacy of the third step of the applied procedure of the mixed monolayer formation. Additionally, further proof of the attachment of the AA photosensitizer to the glassy surface is the presence of the S2p line in XPS spectrum of MIX_TC_APTES@glass. The decomposition of the registered S2p region (Figure 3c) gives S2p_3/2_ component at 168.0 eV with its spin-orbit coupling counterpart, S2p_1/2_, that can be assigned to the sulfur atom present in the central ring of AA. The slightly higher than expected energy of S2p_3/2_ [41] may be connected to the charging of the phenothiazine ring.

#### 3.1.2. Raman Spectroscopy

**Figure 4:**
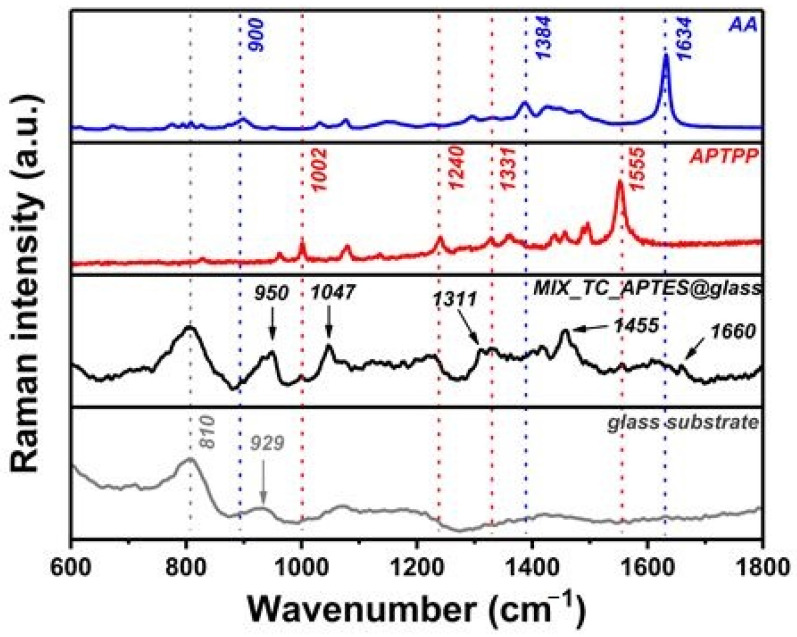
Raman spectra recorded for AA, APTPP, modified (MIX_TC_APTES@glass), and unmodified glass substrate.

The final check of the chemical structure of the deposited photoactive mixed layer was done by Raman spectroscopy. The Raman spectra of the two photosensitizers (AA and APTPP), the MIX_TC_APTES@glass layer, and the unmodified glass (Figure 4) were recorded with the 514 nm laser. The spectrum recorded for MIX_TC_APTES@glass shows signals coming from the inorganic glass substrate and the immobilized organic layer. The broadband located at 810 cm^−1^ arises from the symmetric stretching of the O–Si–O bond, while the signal at 929 cm^−1^ comes from the Si–O stretching vibrations of the glass substrate [42]. The second one is not clearly seen in the spectrum of MIX_TC_APTES@glass but probably leads to an overall increase in the intensity of the modes observed in the 900–950 cm^−1^ range. The presence of the chemically grafted APTES molecules in the deposited layer is confirmed by specific signals at 950, 1047, and 1455 cm^−1^ observed in the spectrum of MIX_TC_APTES@glass, which can be assigned to the vibrations of CH_2_, the skeletal stretching, and the bending vibrations of the CH_2_ units attached to Si atom, respectively [43,44]. The formation of the amide groups with TC linkers in the postmodification steps is supported by the occurrence of the C–N stretching together with the N–H bending at 1311 cm^−1^ and the C=O stretching vibration mode at 1660 cm^−1^ [45].

Finally, the characteristic signals of both immobilized photosensitizers are present in the acquired Raman spectrum of the mixed photoactive layer (see the Raman spectrum of MIX_TC_APTES@glass layer presented in the limited range in Figure S1. For AA, the signals arising from the C–S–C vibrations, C–N–C stretching vibrations, and the C–C stretching within the phenothiazine ring are observed at 900, 1384, and 1634 cm^−1^, respectively [46]. The specific bands coming from APTPP are located at 1002, 1240, 1331, and 1555 cm^−1^ and correspond to the in-plane bending deformation of the phenyl rings, the C_m_–H bending, the symmetric stretching of the pyrrole half-ring, and the symmetric stretching of C_β_–C_β_, respectively [47,48].

### 3.2. Reactive Oxygen Species (ROS) Photogeneration by the Chemically Grafted Mixed Monolayer

As in our previous works [49,50], α-terpinene was used as a chemical trap for the indirect detection of ROS by means of UV–Vis spectroscopy. Since α-terpinene is more stable under white-light illumination than other more common testing systems, such as DPBF in methanol, it was selected as a trap for this study.

**Figure 5:**
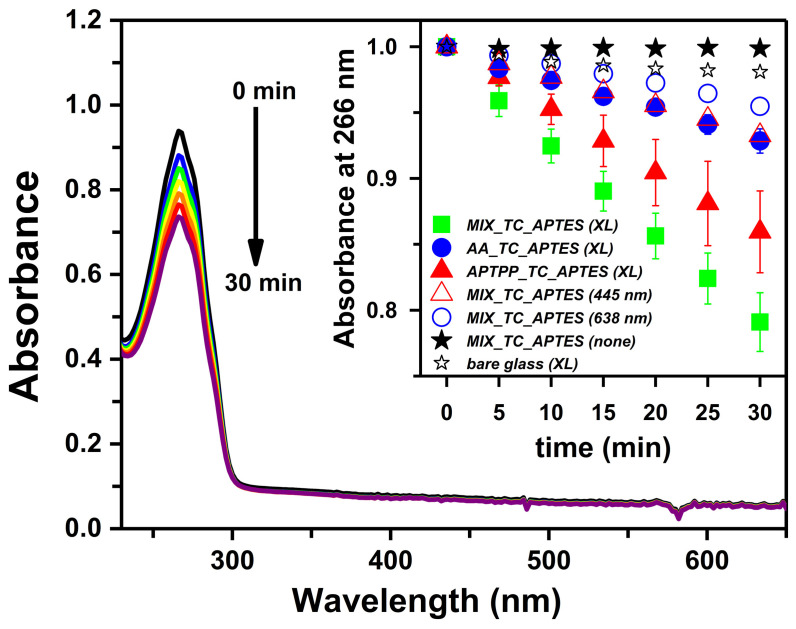
The set of UV–Vis spectra of α-terpinene (ACN solution) registered in the presence of AA_TC_APTES@glass illuminated by the xenon lamp; inset: the absorbance of α-terpinene at 266 nm during illumination of MIX_TC_APTES@glass (**green square**), AA_TC_APTES@glass (**blue circle**), APTPP_TC_APTES@glass (**red triangle**) monolayers, and bare glass (**empty star**) with Xenon lamp (XL); and MIX_TC_APTES@glass monolayer without illumination (**black star**) or under illumination with 445 nm (**empty triangle**) or 638 nm lasers (**empty circle**).

Figure 5 presents an exemplary UV–Vis spectrum recorded for the α-terpinene solution in acetonitrile in contact with the MIX_TC_APTES@glass coating during illumination with the xenon lamp (XL). The clear decrease in the α-terpinene absorbance at 266 nm with time indicates its oxidation by ROS species being produced by the MIX_TC_APTES@glass monolayer. Notably, under applied conditions, only a small drop in the absorbance of α-terpinene, due to its autooxidation, was observed when the unmodified glass was irradiated (Figure 5 inset). As no new bands appear in the UV–Vis spectra at approximately 400 or 600 nm, the degradation of the photoactive layer, resulting in the release of the bound photosensitizers into the solution of the chemical trap, can be excluded. The consistency of the results obtained for the ROS production by the MIX_TC_APTES@glass monolayer under xenon lamp illumination indicates the reproducibility of the layer formation process. Moreover, almost no changes in the UV–Vis spectra during the so-called dark test were detected (Figure 5 inset), which confirms that ROS are produced only when the chemically grafted photosensitizers, i.e., AA and APTPP, are activated by the light.

The photogeneration process was also investigated under the illumination with the 445 or 638 nm diode laser. Use of the blue laser allows to selectively excite APTPP molecules present in the MIX_TC_APTES@glass layer, while the red laser acts mainly on AA (UV–Vis of photosensitizers given in Figure S2). In both tests, a steady drop in the absorbance of α-terpinene at 266 nm occurred (Figure 5 inset) showing that both photosensitizers composing the MIX_TC_APTES@glass layer, retain their photoactivity toward ROS production after immobilization on the glass substrate.

Importantly, in the photogeneration process activated with the xenon lamp, the drop in the α-terpinene concertation observed for the MIX_TC_APTES@glass coating is significantly higher than in the case of the single-component monolayers, i.e., AA_TC_APTES@glass or APTPP_TC_APTES@glass (Figure 5 inset). This proves that in the case of the two-component layer, thanks to the complementarity of the spectral properties of the immobilized photosensitizers, a broader range of light wavelengths can be effectively utilized in the production of the antimicrobial reactive oxygen species.

### 3.3. Antimicrobial Properties of the Chemically Grafted Mixed Monolayer

**Figure 6:**
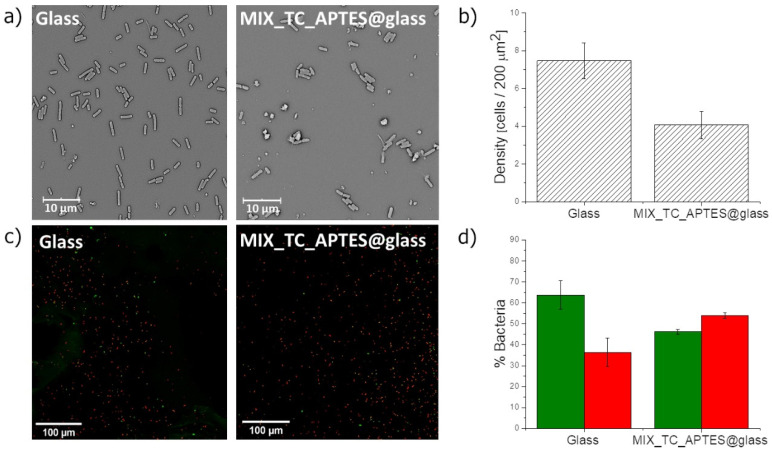
(**a**) SEM images showing *E. coli* present on the unmodified glass surface and MIX_TC_APTES@glass after 3 h; (**b**) the density of bacterial cells after 3 h determined from SEM for the unmodified glass and MIX_TC_APTES@glass surfaces; *p* < 0.05, *n* = 3; (**c**) confocal fluorescent microscope images showing *E. coli* on the unmodified glass and MIX_TC_APTES@glass surfaces after 3 h; (**d**) live and dead bacteria percentages after 3 h for the unmodified glass and MIX_TC_APTES@glass surfaces; *p* < 0.05, *n* = 8.

The bactericidal activity of the MIX_TC_APTES@glass monolayer has been investigated against a Gram-negative *E. coli* strain, which is a common cause of nosocomial infections [51,52,53]. The bacterial culture was carried out under conventional indoor lighting, and the samples for the analysis were collected after 3 and 48 h. The choice of the second time point was based on the possibility of monitoring the prolonged antibacterial action of the investigated layers [54]. SEM was chosen for monitoring the bacterial growth, since it allows to conduct detailed quantitative and qualitative analysis, and thus the influence of the deposited layer on the bacterial cells structure, size, shape, etc. was observed [55]. The average number of bacteria adhering to the surfaces assessed using SEM microscopy and the corresponding calculated densities are shown in Figure 6a,b, respectively. The bacterial viability analysis performed by the Live/Dead assay allowed observation of live (green color) and dead (red color) cells on the surfaces of the MIX_TC_APTES@glass observed with a fluorescent microscope (Figure 6c). The bacterial cells were counted using the ImageJ (FIJI) software and are presented on a graph (Figure 6d).

The bacterial adhesion results observed after 3 h of culture indicate a significantly lower (45.3%) number of the bacterial cells adhered on the MIX_TC_APTES@glass (4.1 ± 0.7 cells/200 µm^2^) compared to the reference unmodified glass slides (7.5 ± 0.9 cells/200 µm^2^). Simultaneously, it is visible that the only 36.3% ± 6.8% of cells present on the unmodified glass during the phase of adhesion are dead, in contrast to cells on the light-activated functionalized surface reaching 53.9% ± 1.2%, which also indicates that the bactericidal action of the layer. Similar results proving the long-term antibacterial character of the MIX_TC_APTES@glass layer were obtained after 48 h of culture (Figure S3). Consequently, as in the SEM micrographs and the same on fluorescent images, the number of live bacterial cells was considerably lower on the light-activated mixed monolayer than on the uncoated glass surface, which confirms the antimicrobial properties of the MIX_TC_APTES@glass coating.

The obtained results confirm that the deposited mixed monolayer, MIX_TC_APTES@glass, possesses antimicrobial properties. Though, the light-activated production of ROS is believed to have the highest contribution to the above-mentioned bactericidal effect [56], it cannot be excluded that the presence of the organic moiety itself influences the adhesion of the bacteria to the surface. Such a beneficial synergic effect, including dark biocide activity [57], has been already reported [58]. It has been already shown that the concentration of the PS in the layer has a high influence on the light-activated antimicrobial efficiency [5,58]. In this work, approximately 0.3 log reduction was obtained for the MIX_TC_APTES@glass nanocoating with respect to the control, i.e., unmodified glass, after 3 h under illumination by the conventional indoor lighting. This is in agreement with the results reported by other groups for coatings with a lower concentration of PS incorporated in the organic matrix [5,59] or for covalently attached PSs [54,60,61,62]. Though, the chemically grafted photoactive layers generally suffer from a lower antimicrobial response, their main advantage is the covalent bond between PS and the surface, which ensures high stability, thus preventing leakage of the PS into the environment (as often observed for the incorporated PS [5]), high adhesion to the surface, and also usually long-term stability. Since such modification occurs at the nanoscale, the coatings usually do not interfere with features such as color, transparency, etc., which is of great importance for the covering of computer screens, windows, etc. Finally, the presented strategy relies on the straightforward processes and commercially available reagents, which makes it easily accessible for the modification of the glassy surfaces regardless of their shape and size.

## 4. Conclusions

In the presented work, AA and APTPP photoactive molecules were chemically grafted on the glass surface in a straightforward three-step procedure. The chemical composition of the deposited layer was confirmed after each step by applying the XPS method, and at the very end, by Raman spectroscopy. The described approach resulted in the formation of the covalently bound mixed monolayer showing photoactivity toward ROS generation. It was shown that the efficiency of the ROS production under white-light illumination of the two-component film is significantly higher than that for the corresponding single-component ones, and that the layer remains inactive in dark conditions. Though, the MIX_TC_APTES layer modifies the glass surface only at the nanoscale, its deposition allows reducing the number of the adhered *E. coli* bacteria, as shown with the microbiological tests. The presented results demonstrate that the undertaken approach allows obtaining a stable, light-activated, antimicrobial nanocoating that may be considered as a promising alternative for the modification of glassy surfaces, where the accumulation of pathogenic material is problematic. The proposed strategy is straightforward and can be applied to various objects regardless their size or shape, without the usage of sophisticated equipment. The strong linkage with the surface atoms ensures that the photoactive molecules will not be released into the environment (as commonly used disinfectants are) and that the layers show long-term antimicrobial response. Finally, such monolayers, due to their nanoscale character, will not interfere with the features, e.g., color, of the modified objects. It is believed that the presented strategy can be further developed using other photosensitizers, in order to increase the broadband absorbance of the layer and, thus, the antimicrobial efficiency.

## Data Availability

Data are presented in the article and Supplementary Materials.

## Supplementary Materials

The following are available online at https://www.mdpi.com/article/10.3390/ma14113093/s1, Figure S1. Raman spectrum of MIX_TC_APTES@glass layer in the range 1350–1700 cm^−1^. Figure S2. UV–Vis spectra of 0.1 mM solution of AA and APTPP, Figure S3. (**a**) SEM images showing *E. coli* present on the unmodified glass surface and MIX_TC_APTES@glass after 48 h; (**b**) the density of bacterial cells after 48 h determined from SEM for the unmodified glass and MIX_TC_APTES@glass surfaces; *p* < 0.05, *n* = 3; (**c**) confocal fluorescent microscope images showing *E. coli* on the unmodified glass and MIX_TC_APTES@glass surfaces after 48 h; (**d**) live and dead bacteria percentages after 48 h for the unmodified glass and MIX_TC_APTES@glass surfaces; *p* < 0.05, *n* = 8.
