# Supplementary material for: Covalent Immobilization of Organic Photosensitizers on the Glass Surface: Toward the Formation of the Light-Activated Antimicrobial Nanocoating
Source: Materials (Basel). 2021 Jun 4;14(11):3093. doi: 10.3390/ma14113093 (PMC8201308; doi:10.3390/ma14113093)
Supplement: Supplementary file 1 [file materials-14-03093-s001.zip › materials-1237221-supplementary.pdf]

materials

MDPI

Supplementary Materials

Covalent Immobilization of Organic Photosensitizers on the Glass Surface: Toward the Formation of the Light-Activated Antimicrobial Nanocoating

Aleksandra Nyga [1], Dominika Czerwińska-Główka [1], Maciej Krzywiecki [2], Wioletta Przystaś [3,4], Ewa Zabłocka-Godlewska [3,4], Sebastian Student [4,5], Monika Kwoka [5,6], Przemysław Data [1] and Agata Blacha-Grzechnik [1,*]

[1] Faculty of Chemistry, Silesian University of Technology, Strzody 9, 44-100 Gliwice, Poland; aleksandra.nyga@polsl.pl (A.N.); dominika.czerwinska-glowka@polsl.pl (D.C.-G.); przemyslaw.data@polsl.pl (P.D.)

[2] Center for Science and Education (CSE), Institute of Physics, Silesian University of Technology, Konarskiego 22B, 44-100 Gliwice, Poland; maciej.krzywiecki@polsl.pl

[3] Faculty of Energy and Environmental Engineering, Silesian University of Technology, 44-100 Gliwice, Poland; wioletta.przystas@polsl.pl (W.P.); ewa.zablocka-godlewska@polsl.pl (E.Z.-G.)

[4] Biotechnology Centre, Silesian University of Technology, 44-100 Gliwice, Poland; sebastian.student@polsl.pl

[5] Faculty of Automatic Control, Electronics and Computer Science, Silesian University of Technology, 44-100 Gliwice, Poland; monika.kwoka@polsl.pl

[6] Institute of Electronics, Silesian University of Technology, Akademicka 16, 44-100 Gliwice, Poland

* Correspondence: agata.blacha@polsl.pl; Tel.: +48-322371024

1. Raman Spectroscopy

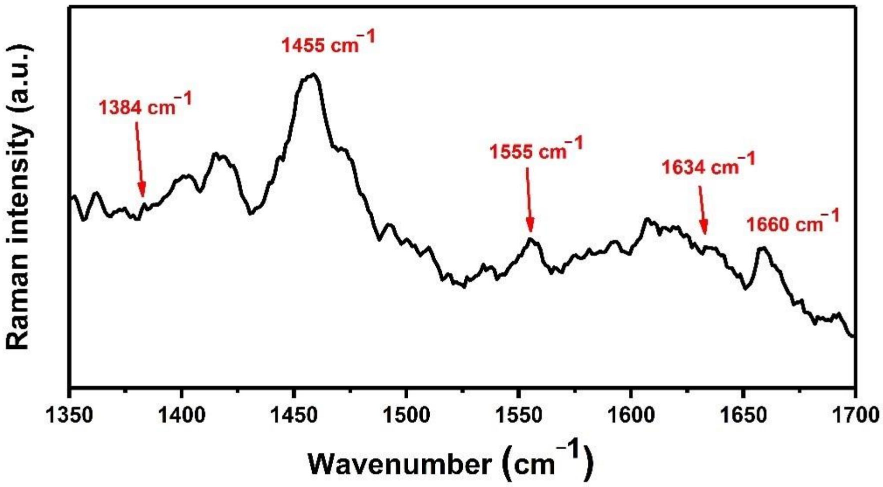

Figure S1. Raman spectrum of MIX_TC_APTES@glass layer in the range 1350–1700 cm^{-1}.

2. UV-Vis Spectroscopy

UV-Vis spectra of 0.1 mM solution of AA and APTPP in in CH_2Cl_2 was recorded by Hewlett Packard (Palo Alto, CA, USA) 8452A UV-Vis spectrometer.

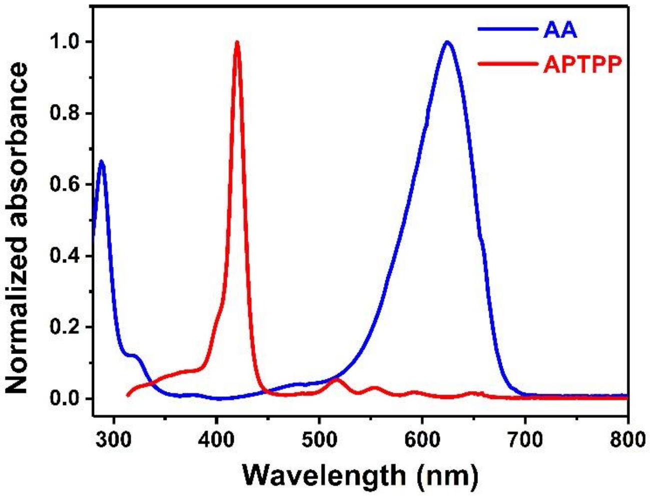

Figure S2. UV-Vis spectra of 0.1 mM solution of AA (**blue line**) and APTPP (**red line**).

3. Antimicrobial tests

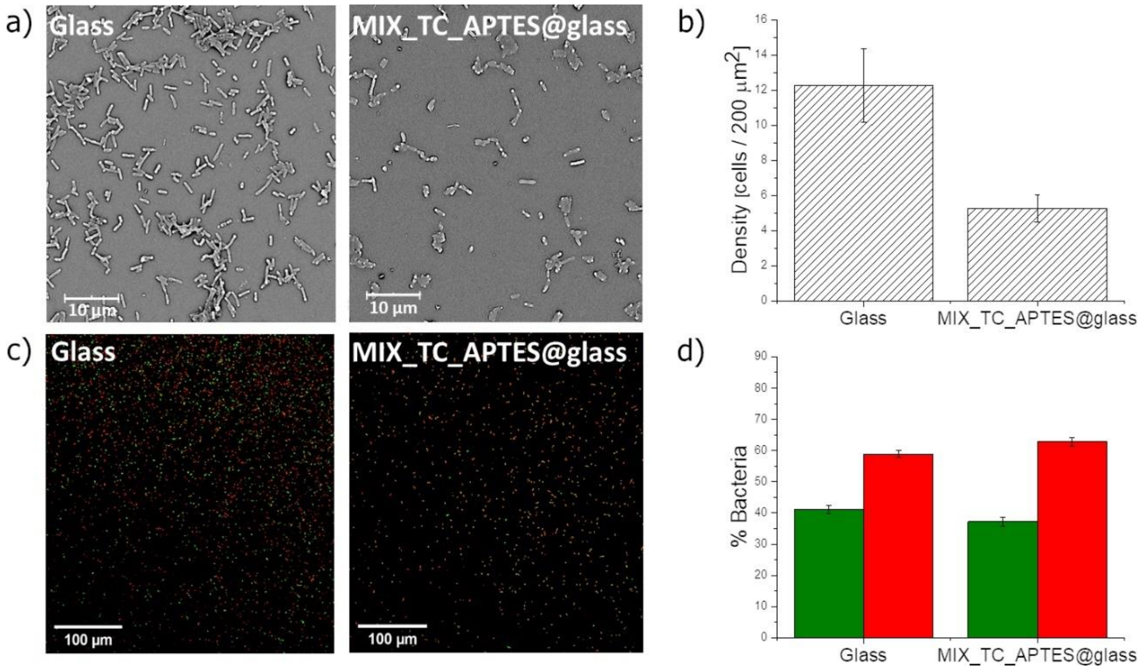

Figure S3. (**a**) SEM images showing *E. coli* present on the unmodified glass surface and MIX_TC_APTES@glass after 48 h; (**b**) the density of bacterial cells after 48 h determined from SEM for the unmodified glass and MIX_TC_APTES@glass surfaces; $p < 0.05$, $n = 3$; (**c**) confocal fluorescent microscope images showing *E. coli* on the unmodified glass and MIX_TC_APTES@glass surfaces after 48 h (**d**) live and dead bacteria percentages after 48 h for the unmodified glass and MIX_TC_APTES@glass surfaces; $p < 0.05$, $n = 8$.
